# Supplementary material for: High Expression of PRNP Predicts Poor Prognosis in Korean Patients with Gastric Cancer
Source: Cancers (Basel). 2022 Jun 28;14(13):3173. doi: 10.3390/cancers14133173 (PMC9264980; doi:10.3390/cancers14133173)
Supplement: Supplementary file 1 [file cancers-14-03173-s001.zip › cancers-1765796-supplementary.pdf]

cancers
MDPI

Supplementary Materials

High Expression of *PRNP* Predicts Poor Prognosis in Korean Patients with Gastric Cancer

Minseok Choi [1,†], SeongRyeol Moon [2,3,†], Hyo Jin Eom [4], Seung Mook Lim [5], Yon Hui Kim [6] and Seungyoon Nam [2,3,7,*]

1 College of Medicine, Gachon University, Incheon 21565, Korea; 201838427@medicine.gachon.ac.kr
2 Department of Health Sciences and Technology, Gachon Advanced Institute for Health Sciences and Technology (GAIHST), Gachon University, Incheon 21999, Korea; moon0620@gachon.ac.kr
3 Department of Genome Medicine and Science, AI Convergence Center for Medical Science, Gachon Institute of Genome Medicine and Science, Gachon University Gil Medical Center, Gachon University College of Medicine, Incheon 21565, Korea
4 Research and Development Department, Corestem Inc., Seongnam 13486, Korea; hyojin3149@gmail.com
5 Department of Biomedical Science, CHA University, Seongnam 13486, Korea; lsmook17@naver.com
6 HieraBio Inc., Seongnam 13605, Korea; sarahkim@hierabio.com
7 Gachon University College of Medicine, Dokjeom-ro 3Beon-gil, 38-13 Namdong-gu, Incheon 21565, Korea
* Correspondence: nams@gachon.ac.kr; Tel.: +82-32-458-2737; Fax: +82-32-458-2875
† These authors contributed equally to this work.

Contents:

Figure S1
Tables S1–S2

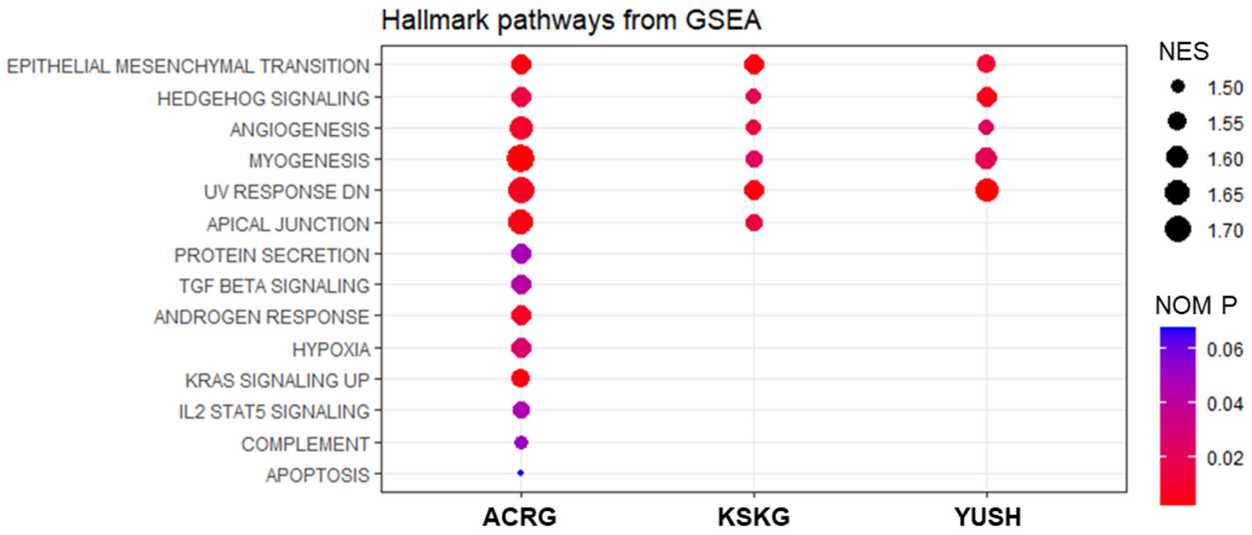

Figure S1. Significantly enriched hallmark pathways in the high PRNP group from GSEA. The size of each dot represents the NES value, and the color represents the P value. It was considered significant when FDR < 0.25. NES, normalized enrichment score.

Table S1. Characteristics of patients from the four gastric cancer cohorts that were recruited.

	ACRG	YUSH	KSKG	TCGA
**N**	300	65	202	377
**Age**				
≥60	183	41	92	261
<60	117	24	110	116
**Sex**				
Male	199	46	142	246
Female	101	19	60	131
**AJCC Stage**				
I	30	12	51	51
II	97	12	36	121
III	96	25	63	168
IV	77	16	51	37
**Lauren classification**				
Intestinal	146	19	141	168
Diffuse	135	30	43	77
NOS	19	17	19	132

NOS, not otherwise specified; AJCC, The American Joint Committee on Cancer.

Table S2. Fold change of differential expression network configuration gene.

Gene	ACRG		KSKG		YUSH	
	FC	P	FC	P	FC	P
PRNP	2.24	< 0.001	3.20	< 0.001	2.31	< 0.001
CSNK2A2	1.03	NS	1.45	< 0.001	1.25	< 0.05
FZD2	1.17	< 0.001	1.70	< 0.001	1.03	NS
FZD6	1.24	< 0.001	1.31	< 0.001	1.40	< 0.01
DVL2	1.01	NS	1.29	< 0.001	1.13	NS
PIK3R1	1.13	< 0.01	1.09	< 0.001	1.15	< 0.001
AKT3	1.25	< 0.001	1.01	NS	1.02	< 0.05
RHOA	1.09	< 0.001	1.12	< 0.001	1.14	< 0.05
PIK3CA	1.24	< 0.001	1.07	< 0.05	1.34	< 0.05
GSK3B	0.95	< 0.05	0.90	< 0.05	0.82	NS

It shows the fold change and P value of the genes constituting the altered sub-pathway gene networks.-NS, not significant.
